# Multicellular Effects of STAT3 in Non-small Cell Lung Cancer: Mechanistic Insights and Therapeutic Opportunities

**DOI:** 10.3390/cancers13246228

**Published:** 2021-12-11

**Authors:** Sagun Parakh, Matthias Ernst, Ashleigh R. Poh

**Affiliations:** 1 Department of Medical Oncology, The Olivia Newton-John Cancer and Wellness Centre, Austin Health, Heidelberg, VIC 3084, Australia; sagun.parakh@onjcri.org.au; 2 Tumor Targeting Laboratory, The Olivia Newton-John Cancer Research Institute, Heidelberg, VIC 3084, Australia; 3 School of Cancer Medicine, La Trobe University, Melbourne, VIC 3086, Australia; matthias.ernst@onjcri.org.au; 4 Cancer and Inflammation Laboratory, The Olivia Newton-John Cancer Research Institute, Heidelberg, VIC 3084, Australia

**Keywords:** STAT3, lung cancer, NSCLC, drug resistance, tumor microenvironment, chemotherapy, immunotherapy, tyrosine kinase inhibitors, clinical trials

## Abstract

**Simple Summary:**

Persistent activation of STAT3 is frequently observed in non-small cell lung cancer and is associated with a poor prognosis. Given the multifaceted role of STAT3 signaling in NSCLC tumor development and progression, this pathway represents a promising therapeutic target for anti-cancer therapy. In this review, we discuss the molecular and immunological mechanisms by which persistent STAT3 activation promotes NSCLC development, and the utility of STAT3 as a prognostic and predictive biomarker. We also provide an update of STAT3-targeting therapies that are currently undergoing Phase I/II clinical trials, and discuss the challenges associated with these treatment modalities in the clinic.

**Abstract:**

Non-small cell lung cancer (NSCLC) is the most common type of lung cancer and accounts for 85% of lung cancer cases. Aberrant activation of the Signal Transducer and Activator of Transcription 3 (STAT3) is frequently observed in NSCLC and is associated with a poor prognosis. Pre-clinical studies have revealed an unequivocal role for tumor cell-intrinsic and extrinsic STAT3 signaling in NSCLC by promoting angiogenesis, cell survival, cancer cell stemness, drug resistance, and evasion of anti-tumor immunity. Several STAT3-targeting strategies have also been investigated in pre-clinical models, and include preventing upstream receptor/ligand interactions, promoting the degradation of STAT3 mRNA, and interfering with STAT3 DNA binding. In this review, we discuss the molecular and immunological mechanisms by which persistent STAT3 activation promotes NSCLC development, and the utility of STAT3 as a prognostic and predictive biomarker in NSCLC. We also provide a comprehensive update of STAT3-targeting therapies that are currently undergoing clinical evaluation, and discuss the challenges associated with these treatment modalities in human patients.

## 1. Non-Small Cell Lung Cancer (NSCLC)

Lung cancer is the most commonly diagnosed cancer and the leading cause of cancer-related deaths worldwide [1]. Non-small cell lung cancer (NSCLC) accounts for 85% of lung cancer cases and can be further divided into three histological subtypes: adenocarcinoma, squamous cell carcinoma, and large cell carcinoma [2]. In NSCLC patients with localized stage I or stage II disease, surgical resection offers a favorable prognosis with a 5-year survival rate of up to 70%. However, a majority (>75%) of patients present with advanced disease at the time of diagnosis and have a 5-year survival rate of less than 25% [3,4,5,6].

Over the past decade, treatment modalities for advanced NSCLC have evolved from traditional chemotherapies such as cytotoxic platinum-based drugs towards more effective regimens that are targeted towards specific molecular subtypes, including those that display epidermal growth factor receptor (EGFR) mutations or anaplastic lymphoma kinase (ALK) fusion oncogenes [7,8]. Although targeted therapies have resulted in clinical benefit, most patients eventually relapse with progressive disease due to the development or selection of new mutations (e.g., EGFR T790M and C797S mutations) or an increase in gene copy number (i.e., ALK oncogene duplication) [9,10,11]. Furthermore, targeted therapies are ineffective in patients that do not have molecularly defined NSCLC, which constitute the majority of lung cancer cases [7,8]. The recent integration of immune checkpoint inhibitors such as antibodies against programmed cell death-1 (PD1), programmed cell death ligand-1 (PDL1), and cytotoxic T-cell lymphocyte antigen-4 (CTLA4) into first-line NSCLC treatment protocols has improved survival and quality of life; however, less than 20% of patients derive long-term benefit and most will eventually succumb to progressive disease during therapy [12,13]. Thus, there is an urgent need to identify additional therapeutic targets to improve treatment outcomes in NSCLC.

## 2. Signal Transducer and Activator of Transcription 3 (STAT3)

The Signal Transducer and Activator of Transcription 3 (STAT3) belongs to the STAT family of proteins, which are both signal transducers and transcription factors. Seven STAT family members have been identified, and include STAT1, STAT2, STAT3, STAT4, STAT5A, STAT5B, and STAT6 [14].

**Figure 1:**
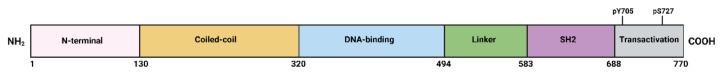
Linear representation of the domain structures of the STAT3 protein. STAT3 is comprised of an N-terminal domain, a Coiled-coil domain, a DNA-binding domain, a Linker, an SH2 domain, and a C-terminal transactivation domain. The C-terminal domain contains a two phosphorylation sites, pY705 and pS727. NH_2_, amino (N) terminus; COOH, carboxy (C) terminus. Figure created in Biorender.

The structure of STAT3 is a characteristic of the STAT family and is comprised of an N-terminal domain, a Coiled-coil domain, a DNA-binding domain, a Linker, an SH2 domain, and a C-terminal transactivation domain (Figure 1) [14,15]. The DNA-binding domain enables the formation of complexes between STAT3 and DNA, and the SH2 domain engages the dimerization of two phosphorylated STAT monomers. Meanwhile, the C-terminal domain contains a tyrosine (Tyr705) residue that is essential for SH2-domain mediated dimerization and hence STAT3 activation, as well as a serine (Ser727) residue which maximizes the transcriptional activity of STAT3 [14,15,16]. Alternative splicing of exon 23 in STAT3 gives rise to truncated versions of STAT3 such as the 770-amino acid STAT3α and 722-amino acid STAT3β isoforms, where a 7-amino acid carboxyl terminus replaces the transcription activation domain [14].

## 3. STAT3 Signaling

**Figure 2:**
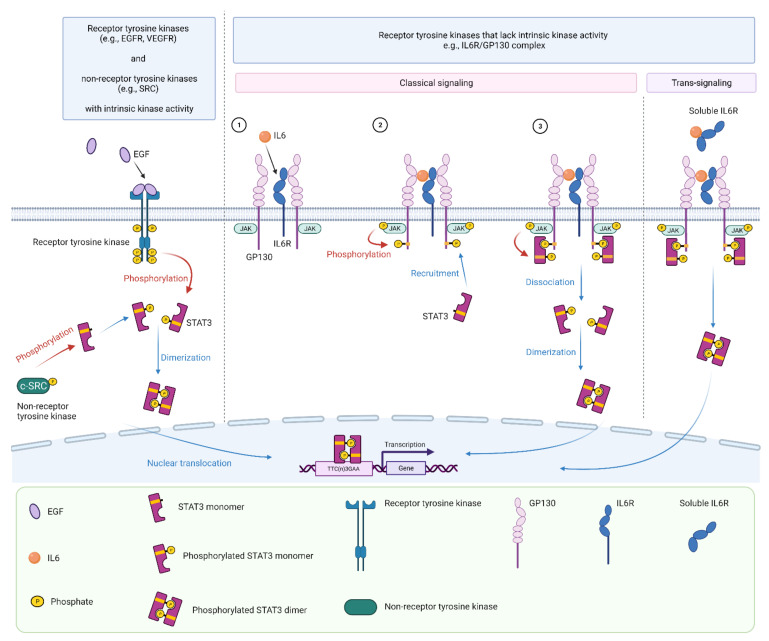
Overview of STAT3 activation. Receptor tyrosine kinases such as EGFR and VEGFR have intrinsic kinase abilities and can directly phosphorylate STAT3 following ligand binding. Receptor independent tyrosine kinases such as c-SRC can also phosphorylate JAK. For receptors that lack intrinsic tyrosine kinase activity, such as the IL6R/GP130 complex, STAT3 activation is initiated upon ligand-receptor interactions (1). Activated JAKs phosphorylate the cytoplasmic tail of the receptor subunit, which serves as a docking site for STAT3 (2). STAT3 is then phosphorylated by JAK, dissociates from the receptor, and forms homodimers that translocate into the nucleus to mediate gene transcription by binding to the TTC(n)3GAA promoter sequence (3). In the alternative trans-signaling pathway, IL6 binds to a soluble IL6 receptor, which then associates with GP130 to initiate downstream STAT3 signaling as per the classical pathway. Figure created in Biorender.

Activation of STAT3 is triggered by a diverse range of cytokines, growth factors, and hormones [17], and can occur via several pathways (Figure 2). Receptor tyrosine kinases such as EGFR and vascular endothelial growth factor receptor (VEGFR) have intrinsic kinase domains and can directly phosphorylate STAT3 following ligand binding [18,19]. Non-receptor tyrosine kinases such as JAKs or c-SRC can also phosphorylate STAT3 without receptor activation [20,21,22].

For receptors that lack intrinsic tyrosine kinase activity, such as the interleukin-6 (IL6) receptor/gp130 complex, activation of STAT3 can occur either via classical or trans-signaling pathways. In the classical signaling pathway, ligand-bound receptors undergo conformational changes that enable the activation of intracellular kinases, including the Janus kinase (JAK) family of non-receptor tyrosine kinases (Figure 2). In turn, JAKs trans-phosphorylate each other and the cytoplastic tail of the receptor subunit, which creates docking sites for STAT3 via its SH2 domain [17]. Upon recruitment, STAT3 is phosphorylated on its C-terminal Tyr705 residue by JAKs. Subsequently, phosphorylated STAT3 dissociates from the receptor complex and forms dimers via binding of the SH2 domain to the phospho-tyrosine residue of another STAT3 or STAT1 molecule. STAT3 homo- and heterodimers are actively transferred to the nucleus where they bind to TTC(n)_3_GAA consensus binding sites in the promoter and enhancer regions of target genes and modulate transcription [17,23].

Certain cytokines such as IL6 and IL11 can also promote STAT3 activation via an alternative trans-signaling pathway [24] (Figure 2). For example, IL6 can bind to a soluble IL6 receptor (sIL6R), which is generated by alternative splicing of IL6R mRNA or cleavage of the membrane-bound IL6R by proteases such as ADAM10 or ADAM17. The IL6-sIL6R ligand-receptor complex then associates with a second protein, gp130, which dimerizes and initiates intracellular signaling as per the classical pathway [24]. Of note, IL6 trans-signaling mediates pro-inflammatory responses whereas classic IL6 signaling via the membrane bound IL6R mediates anti-inflammatory responses [24].

In normal cells, STAT3 activation is tightly regulated by several distinct mechanisms, including protein tyrosine phosphatases (PTPs), protein inhibitors of activated STATs (PIAS), and suppressors of cytokine signaling (SOCS) [25,26]. PTPs such as PTPRD and PTPN11 negatively regulate STAT3 signaling by dephosphorylating its tyrosine residue [27], while PIAS3 binds to STAT3 and interferes with its ability to activate gene transcription [28]. In contrast, SOCS3 disrupts STAT3 signaling by directly inhibiting JAK activity, by competing with STAT3 for phosphotyrosine residues on receptor chains, and by binding to signaling proteins to trigger their proteasomal degradation [29,30].

## 4. Tumor-Promoting Effects of STAT3 Activation in NSCLC

STAT3 is persistently activated in over 50% of NSCLC patients [31,32], and its increased expression is associated with poor tumor differentiation, advanced clinical stage, lymph node metastasis, and drug resistance [33,34,35]. Mutations in receptor tyrosine kinases such as EGFR, as well as in the SRC family of non-tyrosine kinases have been implicated for constitutively activated STAT3 signaling in NSCLC [35,36]. Increased levels of STAT3-activating cytokines (e.g., IL6, IL11, IL22) and growth factors (e.g., HGF, leptin) are also observed in the serum and tumors of patients with NSCLC and promote persistent STAT3 activation via autocrine and paracrine mechanisms. Additionally, disruption of STAT3 regulators such as PTP, PIAS, or SOCS proteins is also observed in NSCLC tumors and results in increased levels of phosphorylated STAT3 [37,38,39]. Importantly, STAT3 is dispensable for the growth and survival of normal cells, which makes it a valuable cancer-specific target [40].

**Figure 3:**
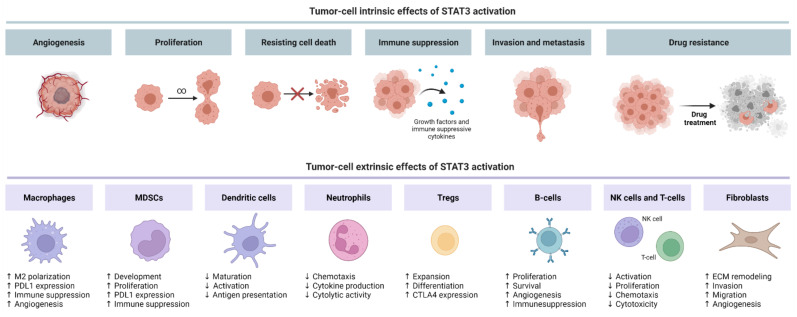
Tumor promoting effects of STAT3 activation in NSCLC. Tumor-cell intrinsic effects of STAT3 activation include angiogenesis, proliferation, immune suppression, invasion, and metastasis. Meanwhile, tumor-cell extrinsic activation of STAT3 in immune and stromal cells favors an immunosuppressive tumor microenvironment that inhibits the activation and recruitment of cytotoxic effector cells. Figure created in Biorender.

To date, the tumor-promoting roles of STAT3 signaling in NSCLC that have been well characterized include promoting angiogenesis, cell survival, cancer cell stemness, drug resistance, and evasion of anti-tumor immunity (Figure 3).

### 4.1. Angiogenesis

Tumor cells rely on the formation of new blood vessels to obtain nutrients and oxygen [41]. This involves a complex process that is tightly regulated by the balance between pro-angiogenic and anti-angiogenic factors [42]. Among them, the vascular endothelial growth factor (VEGF) is a key mediator of blood vessel formation, and numerous studies have demonstrated a pivotal role for tumor cell-intrinsic STAT3 signaling in promoting angiogenesis in NSCLC by upregulating the expression VEGF and other growth factors such as basic fibroblast growth factor (bFGF) [43,44,45,46]. Likewise, tumor cell-extrinsic activation of STAT3 in immune cells such as B-cells and MDSCs enhances angiogenesis and the growth of lung cancer allografts in mice [47,48]. Accordingly, high STAT3 expression is associated with increased microvessel density and poor survival in NSCLC patients [45].

Inhibition of STAT3 in human PC14PE6/AS2 lung adenocarcinoma cells reduces tumor-associated VEGF expression, angiogenesis, and vascular permeability [49], while pharmacologic inhibition or siRNA-mediated interference of JAK2/STAT3 signaling in A549 NSCLC cells impairs angiogenesis by decreasing VEGF expression [45,50]. Dysregulated activation of the STAT3/miR-135b/NFκB signaling axis also contributes to progression of NSCLC by enhancing cell migration, invasion, and angiogenesis [51]. Additionally, a miR-199a-5p/HIF1α/STAT3 positive feedback loop promotes resistance of NSCLC cells to the anti-angiogenic drug bevacizumab [52], while miR-206 decreases angiogenesis in A549 xenografts by inhibiting the STAT3/HIF1α/VEGF pathway [45]. Collectively, these findings demonstrate the pivotal role of STAT3 in regulating angiogenesis in NSCLC.

### 4.2. Cell Survival

Persistent activation of STAT3 also contributes to tumorigenesis by upregulating the transcription of genes that control cancer cell survival (e.g., cyclins and c-MYC [53,54]), resistance to apoptosis (e.g., BCL2, MCL1 [55]), and cell cycle activation [56]. Constitutive STAT3-binding activity is observed in most human NSCLC cell lines and promotes tumor cell proliferation in response to growth factors (e.g., EGF, HGF) and cytokines (e.g., IL6) [57]. This interaction is dependent on SRC-kinase activity since therapeutic inhibition of SRC abrogates STAT3 binding and induces cell-cycle arrest and apoptosis. Conversely, disruption of STAT3 with anti-sense oligonucleotides or an adenoviral vector expressing a dominant-negative form of STAT3 promotes apoptosis of NSCLC cells [57]. STAT3 also contributes to the survival of EGFR-mutant NSCLC cells by enhancing their proliferative capacity, while therapeutic inhibition of STAT3 triggers apoptosis [58,59,60]. Collectively, these findings highlight the versatile role of STAT3 signaling in regulating cell survival and apoptosis in NSCLC.

### 4.3. Promoting Cancer Cell Stemness

Cancer stem cells (CSCs) are a subpopulation of tumor-initiating cells [61], and their increased abundance is associated with a poor outcome in NSCLC by contributing to drug resistance and disease recurrence [62,63,64]. Two distinct NSCLC cancer stem cell populations have been identified by expression of aldehyde dehydrogenase (ALDH) and CD133 [65]. ALDH1^high^ CSCs express higher STAT3 levels than ALDH1^low^ cells, and are more tumorigenic and resistant to gamma-radiation and cytotoxic drugs [66]. CD133^+^ CSCs also exhibit greater tumorigenicity and chemo/radio-resistance than CD133^−^ CSCs [67], which correlates with increasing levels of STAT3 activation [68]. Notably, the potent STAT3 inhibitor, cucurbitacin I, suppresses the self-renewal and proliferative phenotype of CD133^+^ CSCs and improves sensitivity to radiotherapy and chemotherapy [68]. Consistent with previous reports demonstrating a role for STAT3 in mediating the epithelial-to-mesenchymal transition (EMT) of lung cancer cells, inhibition of STAT3 by cucurbitacin I also inhibits the migratory and metastatic ability of CD133^+^ CSCs in vivo [68,69]. In another study, the small molecule STAT3 inhibitor BBI-608 was found to inhibit stemness gene expression and deplete CSCs in a pre-clinical model of NSCLC [70]. Thus, these findings support a central role for STAT3 in maintaining the malignant phenotype of tumor initiating cells.

### 4.4. Drug Resistance in Oncogene-Addicted Cells

‘Oncogene-addicted’ cancer cells are dependent on a single activated oncogenic protein or pathway to maintain their malignant phenotype. Although they can be targeted by pathway-specific inhibitors, long-term response to therapy is inevitably limited by the emergence of drug-resistant cells [71]. Feedback activation of STAT3 in oncogene-addicted cancer cells has been implicated in promoting drug resistance and can be inhibited to restore drug sensitivity [35].

Despite initial dramatic responses to EGFR tyrosine kinase inhibitors in patients with activating EGFR mutations (e.g., exon 19 in-frame deletions or exon 21 L858R point mutation), almost all patients eventually relapse due to acquired resistance mechanisms. In 50% of patients, resistance is driven by a secondary T790M mutation in exon 20 of EGFR [72,73]. Aberrant activation of the IL6R/JAK1/STAT3 signaling pathway is implicated in mediating de novo resistance to tyrosine kinase inhibitors (e.g., afatinib, erlotinib, dacomitinib) in H1975 and PC9-GR NSCLC cells harboring the T790M mutation [36]. Mechanistically, afatinib and dacomitinib induce STAT3 activation via promoting autocrine IL6 secretion in cancer cells, which potentiates drug resistance via a paracrine loop between fibroblasts and tumor cells [36]. Accordingly, pharmacologic targeting of STAT3 overcomes resistance to afatinib and enhances anti-tumor immunity PC9-GR tumor-bearing mice [36].

In another study, exposure of treatment naïve EGFR-mutant PC-9 NSCLC cells to conditioned media of erlotinib-treated cells also enhanced drug resistance in a STAT3-dependent manner [35]. In contrast, STAT3 depletion via RNAi enhanced tumor cell apoptosis following erlotinib treatment, while transient STAT3 knock-down suppressed colony formation in drug-resistant cells [35]. These findings were extended to KRAS-driven NSCLC, where resistance of KRAS-mutant NSCLC cells to MEK inhibitors was reversed upon inhibition of STAT3 activity [35]. Collectively, these findings support a role for feedback activation of STAT3 in limiting the overall drug response of oncogene-addicted NSCLC.

### 4.5. Immune Modulation via Tumor-Cell Intrinsic STAT3 Activation

The tumor microenvironment of NSCLC is comprised of a heterogenous population of cells, including T-cells, B-cells, dendritic cells, natural killer (NK) cells, myeloid-derived suppressor cells (MDSCs), neutrophils, and macrophages. While the contribution of the immune microenvironment in NSCLC has been extensively reviewed by others [74,75], NSCLC patients with metastasis present with systemic anti-tumor immune deficiency [74]. The efficacy of immune checkpoint inhibitors such as anti-PD1 immunotherapy in NSCLC also highlights the importance of disrupting the immunosuppressive microenvironment to inhibit tumor growth [76].

Several studies have highlighted the role of tumor-cell intrinsic STAT3 signaling in modulating the tumor microenvironment of NSCLC [77,78,79,80,81]. Overexpression of STAT3 in alveolar type II epithelial cells leads to severe pulmonary inflammation associated with extensive immune cell infiltration and upregulation of proinflammatory cytokines and chemokines in the lung [77]. This results in impaired immune surveillance and the upregulation of genes that stimulate epithelial cell growth. Consequently, persistent STAT3 activation induces the development of spontaneous lung bronchoalveolar adenocarcinoma in mice [77]. The role of tumor-cell intrinsic STAT3 in cancer-associated inflammation has also been investigated in a urethane-induced model of lung adenocarcinoma, where epithelium-specific ablation of STAT3 significantly reduces carcinogen-induced lung tumorigenesis [78]. These changes were associated with increased proinflammatory chemokine production and enhanced NK cell immunity. In addition, STAT3-silenced human NSCLC cells display an enhancement of proinflammatory chemokine production and increased susceptibility to NK cell-mediated cytotoxicity [78]. Collectively, these findings demonstrate an inhibitory effect of STAT3 signaling on anti-tumor NK immunity in carcinogen-induced tumors.

Tumor cell-intrinsic STAT3 can also exert inhibitory effects on cytotoxic T-cell responses through the upregulation of immune checkpoint molecules such as PDL1. For example, aberrant EGFR signaling in NSCLC cells upregulates PDL1 expression through activation of the IL6/JAK/STAT3 pathway [80], while combined STAT3 and PDL1 inhibition renders tumor cells more susceptible to cytotoxic T-cell mediated killing and delays tumor growth in mice [81].

Persistent activation of STAT3 in tumor epithelial cells also promotes the secretion of pro-inflammatory cytokines, growth factors, and chemokines that facilitate the recruitment of immunosuppressive myeloid cells into tumors. In turn, these cells perpetuate an immunosuppressive tumor microenvironment via a feedforward loop through the secretion of pro-tumorigenic factors that sustain STAT3 activation within tumor cells [82,83,84].

### 4.6. Immune Modulation via Tumor-Cell Extrinsic STAT3 Activation

STAT3 is constitutively activated in tumor-infiltrating immune cells, and genetic ablation of STAT3 in these cells unleashes an intrinsic immune-surveillance system that abrogates tumor growth and metastasis in mice [85]. Moreover, aberrant activation of STAT3 in myeloid cells promotes lung cancer by facilitating the recruitment of immunosuppressive cell types (e.g., regulatory T-cells (Tregs), MDSCs, alternatively activated macrophages) into the tumor microenvironment, while myeloid-specific deletion of STAT3 in mice unleashes anti-tumor immunity by enhancing cytotoxic T- and NK cell responses [48]. Here, we expand on the tumor cell-extrinsic role of STAT3 signaling in the tumor microenvironment by modulating the activity of immune and stromal cells.

Macrophages—Macrophages are a major component of solid cancers including NSCLC and accumulate during early tumor development to promote EMT and tumor cell invasion [86]. Macrophages can reversibly alter their endotype in response to environmental cues and are broadly classified into classically or alternatively activated subtypes [87]. Classically activated (M1-like) macrophages secrete inflammatory cytokines (e.g., IL1, IL6, and TNFα) and effector molecules (including reactive nitrogen intermediates) that enhance the activation of cytotoxic effector cells [87]. In contrast, alternatively activated (M2-like) macrophages exhibit an immune suppressive and pro-angiogenic endotype and contribute to tumor development by facilitating immune evasion and escape. STAT3 acts as a key regulator of alternatively activated macrophages in pre-clinical models of lung cancer [88,89,90,91], and also induces surface expression of PDL1 to suppress anti-tumor immune responses [92]. Meanwhile, STAT3 ablation in macrophages promotes the polarization of classically activated macrophages, prevents T-cell tolerance, and augments cytotoxic T- and NK cell responses [48,93,94,95]. In line with these observations, macrophages isolated from conditional STAT3 knock-out mice also demonstrate an enhanced ability to prime and cross-present tumor-derived antigens to cytotoxic T-cells [96].

Myeloid-derived suppressor cells (MDSCs)—MDSCs are a heterogenous population of cells that have emerged as a major regulator of immune responses, and their increased abundance is associated with disease progression and poor clinical outcome in NSCLC [97]. STAT3 is a key transcriptional regulator of MDSC function and expansion [98] and is significantly upregulated in MDSCs of tumor-bearing mice compared to immature myeloid cells in naïve mice [99]. In pre-clinical models of lung cancer, STAT3 promotes MDSC development and proliferation, and induces the expression of key pro-apoptotic mediators to kill cytotoxic T-cells. Persistent activation of STAT3 in MDSCs also induces the expression of immunosuppressive cytokines such as IL10, TGFβ, and NOX2, which promotes Treg development and inhibits dendritic cell activation [100,101]. Conversely, ablation of STAT3 in myeloid cells including MDSCs suppresses lung tumorigenesis in mice by reinvigorating anti-tumor immunity [100]. Of note, alternatively activated macrophages and MDSCs are a major source of STAT3-activating cytokines such as IL6 and IL11, which form a paracrine loop to perpetuate a tumor-reactive microenvironment by acting on cancer cells [87,102,103].

Dendritic cells—Dendritic cells play a pivotal role in mediating protective immunity in lung cancer by facilitating the activation of antigen-specific CD8 T-cells [104,105]. STAT3-activating cytokines (e.g., IL6, IL10, VEGF) secreted by tumor cells promote abnormal dendritic cell differentiation and globally suppress dendritic cell maturation and activation [106,107,108,109,110,111]. Meanwhile, STAT3-deficient dendritic cells demonstrate enhanced immune activity, including increased cytokine production, antigen-dependent T-cell activation, and resistance to IL10-mediated suppression [109].

Neutrophils—Neutrophils correlate with increased tumor burden in NSCLC patients and are associated with reduced T-cell responses, decreased T-cell infiltration, and diminished expression of IFNγ-related genes [112]. Numerous studies have highlighted a role for neutrophils in driving oncogenic transformation in lung cancer cells by promoting DNA damage through the release of reactive oxygen species [113], while direct cell–cell interactions between neutrophils and tumor cells promotes the release of inflammatory mediators that enhance tumor growth in NSCLC [114]. STAT3 plays a central role in neutrophil biology by regulating the production of inflammatory cytokines (e.g., IL1, IFNγ, TNF) [115,116], while STAT3 inhibition in neutrophils enhances their cytolytic activity and promotes tumor regression [85].

Natural killer cells—NK cells are a class of innate lymphoid cells that play an important role in inflammation, antigen presentation, and adaptive immune responses [117]. Numerous studies have highlighted a role for STAT3 as a negative regulator of NK cell activity, since loss of STAT3 in NK cells enhances NK cell-dependent tumor surveillance across different cancer types [83,85,118,119]. In a carcinogen-driven model of NSCLC, genetic ablation of STAT3 in mice reduced urethane-induced tumorigenesis and increased anti-tumor inflammation by enhancing NK cell recruitment and activation [78]. In line with these observations, STAT3 overexpression in NK cells abrogates NK cytotoxic effector functions, while targeted inhibition of STAT3 improves NK-mediating killing of NSCLC cells in vitro [120].

B-cells—STAT3 positively regulates B-cell development, maturation, and proliferation, while genetic ablation of STAT3 in mice reduces the number of mature B-cells in the bone marrow and periphery [121]. STAT3 signaling in B-cells is also essential for germinal center formation and maintenance, as well as antibody responses [122]. STAT3 activation in B-cells enhances tumor angiogenesis via upregulation of VEGF in lung cancer allografts [47] and increases surface expression of immune checkpoint molecules such as CTLA4 [123]. Regulatory B-cells that express high levels of STAT3 have also been identified in the draining lymph nodes of lung cancer patients and promote tumor progression by inducing angiogenesis and immunosuppression via production of IL10 [124,125]. Of note, inactivation of STAT3 in regulatory B-cells reduces IL10 and TGFβ production, and augments anti-tumor immunity by enhancing cytotoxic T-cell activity and by decreasing the number of Tregs in draining lymph nodes and tumor tissues [126].

Tregs—Tregs are a subset of CD4 T-cells that sustain an immunosuppressive tumor microenvironment via secretion of IL10 and TGFβ [127,128]. In turn, TGFβ induces the transcription of Forkhead box P3 (FOXP3), which converts naive CD4 T-cells into Tregs [129]. STAT3 can bind directly to the transcriptional promoter of FOXP3 to induce its expression [130,131,132] or by binding to the promoter of TGFβ and IL10 in Tregs [133]. Moreover, IL10R-mediated STAT3 signaling enhances the expression of CTLA4 on the surface of Tregs and augments their suppressive capability [134]. Conversely, therapeutic targeting of STAT3 enhances anti-tumor T-cell cytotoxicity and reverses immune suppression by abrogating Tregs [135]. Inhibiting STAT3 in hematopoietic cells also significantly reduces the number of FOXP3 Tregs and promotes CD8 T-cell proliferation [85]. Together, these findings suggest a critical role for STAT3 in the functional maintenance of Tregs.

T-cells—T-cells are critical in eliciting anti-tumor responses, and a high density of CD8 T-cells in the peripheral blood [136] and tumor stroma [137,138] of NSCLC patients is associated with a favorable prognosis. However, most T-cells in NSCLC patients exhibit an exhausted endotype characterized by diminished production of inflammatory cytokines and impaired cytotoxicity [139,140]. STAT3 has been shown to directly exert immune suppressive effects on T-cells by inhibiting their recruitment, proliferation, and survival [141,142,143], and influences immune tolerance by regulating the expression of immune checkpoint proteins such as PD1 and CTLA4 on the surface of these cells [144,145]. Meanwhile, genetic ablation of STAT3 in CD8 T-cells enhances their infiltration into tumors, promotes their proliferation, and results in increased cytotoxic T-cell activity and tumor growth inhibition [146].

Fibroblasts—Cancer-associated fibroblasts (CAFs) are a key component of the tumor microenvironment and play a pivotal role in immune suppression, extracellular matrix deposition, and remodeling [147]. The JAK/STAT signaling pathway is constitutively activated in CAFs, and fibroblast-derived cytokines (e.g., IL6, IL10, IL11, IL22) act as ligands to amplify the JAK/STAT signaling cascade [148]. CAFs enhance the metastatic potential of human NSCLC cells through a IL6/STAT3 signaling pathway [149], which in turn promotes tumor angiogenesis through the upregulation of VEGF and bFGF [45]. Accordingly, inhibition of the IL6/STAT3 signaling pathway using an IL6 neutralizing antibody or JAK2/STAT3 inhibitor reverses fibroblast-induced invasion and migration of lung cancer cells [149]. Persistent activation of STAT3 in CAFs also directly enhances their pro-angiogenic, migratory, and invasive phenotype [150,151,152], which can be therapeutically inhibited using small molecule STAT3 inhibitors such as Stattic [153]. Of note, mesenchymal stem cells in the tumor microenvironment can also be induced to differentiate into CAFs through activation of the JAK/STAT3 signaling cascade [154].

## 5. Role of STAT3-Activating Cytokines and Growth Factors in NSCLC

Numerous STAT3-activating cytokines and growth factors are significantly elevated in NSCLC patients and are directly associated with a poor clinical outcome. Here, we discuss the contribution of these cytokines and growth factors in NSCLC via downstream activation of STAT3.

IL6—IL6 is highly expressed in the serum and breath condensate of NSCLC patients and is associated with increased risk of metastasis and chemoresistance [155,156,157,158,159,160,161,162]. In a CCSP^Cre^/LSL-Kras^G12D^ mouse model of KRAS-driven NSCLC, IL6 induced the proliferation of tumor cells and facilitated an immunosuppressive microenvironment by enhancing the polarization of alternatively activated macrophages and recruitment of MDSCs [103]. Conversely, IL6 blockade inhibited epithelial STAT3 activation and tumor growth in mice [18,103,163,164]. Consistent with the role of IL6 in modulating the tumor microenvironment of NSCLC, patients with high levels of circulating IL6 also display more immunosuppressive Tregs than patients with low IL6 levels [165] and exhibit poorer response to anti-PD1/PDL1 immune checkpoint blockade [166,167]. In line with these observations, IL6 blockade significantly reduced tumor development in a KRAS-mutant mouse model of lung cancer by downregulating tumor cell-intrinsic STAT3 activation, tumor cell proliferation, and the expression of angiogenesis markers. IL6 inhibition also reduced the abundance of alternatively activated macrophages, MDSCs, and augmented cytotoxic T-cell responses [103].

Studies conducted in the Gp130^F/F^;Kras^G12D^ model of lung adenocarcinoma have also revealed a role for IL6 trans-signaling in KRAS-driven lung tumorigenesis [168]. In Gp130^F/F^ mice, a phenylalanine knock-in substitution at tyrosine 757 in the cytoplasmic domain of Gp130 prevents binding of SOCS3 and facilitates excessive activation of STAT3 [169]. Accordingly, increased levels of soluble IL6R are observed in the lungs of tumor-bearing Gp130^F/F^;Kras^G12D^ mice, and blocking this signaling pathway with an anti-IL6R antibody or the inhibitor sgp130Fc abrogates tumorigenesis in vivo [168].

IL6 has also been shown to promote EMT, tumor cell invasion, and drug resistance to tyrosine kinase inhibitors in NSCLC [170,171,172,173]. Interestingly, IL6 contributes to radiation-induced macrophage migration in NSCLC, which has been shown to accelerate tumor progression [174]. Furthermore, IL6 is a potential mediator of immune-related adverse events in NSCLC patients treated with immune checkpoint blockade [175] and chemoradiation therapy [176,177].

IL10—IL10 is a potent anti-inflammatory cytokine that is produced by almost all leukocytes [178] and correlates with a poor prognosis in NSCLC patients [179,180]. Increased levels of IL10 are also detected in the tumors and serum of genetically engineered EGFR^L858R^ and Kras4b^G12D^ mice that spontaneously develop lung adenocarcinomas [179]. IL10 was observed to activate JAK/STAT3 signaling and induce EGFR expression, which in turn promoted IL10 expression via PI3K/nucleolin signaling to facilitate a feedforward loop that supports tumor development. Meanwhile, genetic ablation of IL10 in EGFR^L858R^ and Kras4b^G12D^ mice impaired lung tumorigenesis by inhibiting EGFR and by decreasing the infiltration of immune-suppressive macrophages and Tregs [179]. The latter observation is consistent with the role of IL10 in regulating immune tolerance in NSCLC patients by increasing Treg infiltration and the expression of immune checkpoint proteins such as PD1 and PDL1 [181].

IL11—Elevated IL11 expression is observed in tumors, serum, and breadth condensate of NSCLC patients, and is associated increased risk of metastasis [182,183]. IL11 enhances tumor cell proliferation, migration, EMT, and invasion of NSCLC cells via activation of STAT3, while depletion of IL11 significantly impairs the growth of NSCLC xenografts in mice and improves survival [182].

IL17—IL17 is a pro-inflammatory cytokine that is primarily produced by activated CD4 T helper cells (Th cells) [184]. Increased IL17 levels correlate with tumor recurrence, metastasis, and poor survival in pre-clinical models and NSCLC patients by enhancing VEGF-mediated angiogenesis in a STAT3-dependent manner [185,186,187,188]. Meanwhile, an IL17-mediated paracrine loop between innate and adaptive immune cells promotes resistance of lung tumors to anti-angiogenic therapies, which can be reversed upon pharmacologic blockade of IL17 [189]. Additional studies have highlighted a role for IL17 in inducing EMT via STAT3 activation, while pharmacologic STAT3 inhibition or siRNA knock-down reduces IL17-induced EMT in A549 NSCLC cells [190].

IL22—IL22 is produced by CD4 T-cells, NK T-cells, innate lymphoid cells, and γδ T-cells, and triggers STAT3 activation upon binding to IL22R1 on epithelial cells [191]. IL22 is upregulated in tumor tissues and serum from patients with recurrent NSCLC compared to primary NSCLC tumors and is associated with poor clinical outcome [192,193]. Elevated IL22 is also observed in NSCLC patients who are resistant to EGFR tyrosine kinase inhibitors, and induces gefitinib resistance in NSCLC cell lines [194]. Treatment of NSCLC cell lines with IL22 enhances proliferation, migration, and invasion via activation of IL22R1/STAT3 signaling, while siRNA-mediated depletion of IL22R1 abrogates the effects of IL122 on cell proliferation and migration [192]. IL22 also plays an essential role in KRAS-mutant lung cancer by inducing an inflammatory tumor microenvironment and protects lung cancer cell stemness [195]. Meanwhile, overexpression of IL22 protects NSCLC cancer cell lines from chemotherapy-induced apoptosis via activation of STAT3, which triggers the expression of downstream anti-apoptotic proteins including BCL2 and BCL-xL [196].

IL26—IL26 is a pro-inflammatory cytokine that is primarily produced by Th17 cells [197]. Increased IL26 expression is observed in the malignant pleural effusion (MPE) and peripheral blood of lung cancer patients, and predicts poor patient survival in part by suppressing CD8 T-cell cytotoxicity [198].

Leukemia inhibitory factor (LIF)—LIF is significantly upregulated in NSCLC tumors compared to adjacent tissues, and correlates with lymph node metastasis and advanced tumor stage [199]. Treatment of NSCLC cell lines with LIF induces cellular proliferation, invasion, and migration in STAT3-dependent manner, which can be rescued following treatment with the STAT3 inhibitor Stattic [199].

Oncostatin M (OSM)—High OSM expression is associated with a poor prognosis in lung cancer patients and facilitates the EMT of NSCLC cells [200]. The OSM pathway also protects NSCLC cells harboring KRAS/EGFR mutations and EML4-ALK fusions from apoptosis via activation of OSMRs/JAK1/STAT3 signaling [200]. Meanwhile, secretion of IL6 and OSM by tumor stromal fibroblasts contributes to resistance of NSCLC cells to targeted therapies [200].

Hepatocyte growth factor (HGF)—HGF induces a wide variety of cellular responses by binding to the MET tyrosine kinase receptor, which results in downstream STAT3 activation. Aberrant HGF/MET signaling is associated with a poorer overall survival in NSCLC patients [157,201,202,203] and contributes to tumorigenesis by facilitating anchorage independent growth of NSCLC cells in a STAT3-dependent manner [204].

Leptin—Elevated expression of the hormone leptin is associated with reduced overall survival in NSCLC [205] and promotes metastasis by inducing EMT in lung cancer cells [206]. Conversely, leptin knock-down impairs NSCLC tumor cell proliferation and induces apoptosis by inhibiting JAK/STAT3 signaling [207].

## 6. STAT3 as a Therapeutic Target

**Figure 4:**
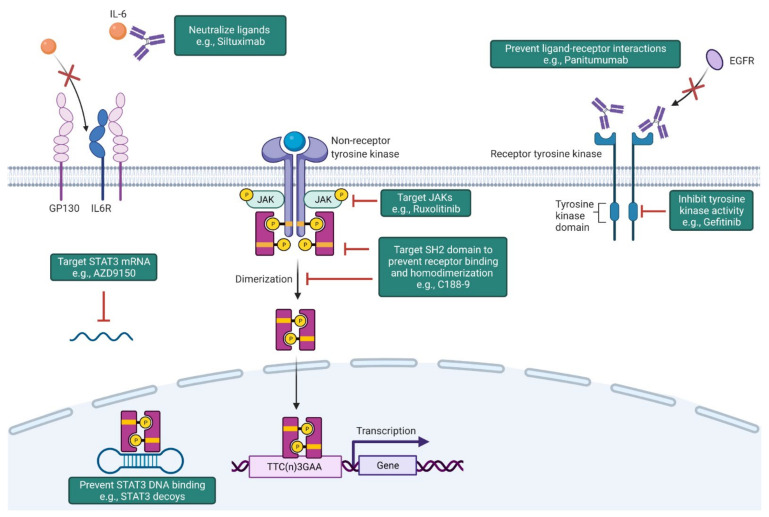
Strategies to inhibit STAT3 activation. Strategies to inhibit STAT3 signaling include preventing receptor/ligand interactions, inhibiting tyrosine kinase activity, targeting the SH2 domain of STAT3 to interfere with receptor binding and/or homodimerization, promoting the degradation of STAT3 mRNA, and blocking the binding of STAT3 to DNA. Figure created in Biorender.

Given its multifaceted role in NSCLC development, progression, and therapy resistance, STAT3 represents an attractive drug target to impair tumor growth. Furthermore, STAT3 is dispensable for the growth and survival of normal cells, which makes it a valuable cancer-specific target [40]. Strategies to inhibit STAT3 include preventing receptor/ligand interactions, targeting the SH2 domain of STAT3, promoting the degradation of STAT3 mRNA, and interfering with STAT3 DNA binding (Figure 4). A number of these approaches have also been evaluated in clinical trials (Table 1).

### 6.1. Targeting Upstream Signaling Mechanisms

Therapeutic approaches to prevent STAT3 activation include targeting upstream signaling mechanisms, including growth factors and cytokines along with their corresponding receptors and tyrosine kinases. Several of these drugs have been evaluated in clinical trials, with mixed results (Table 1).

Inhibiting the IL6 signaling cascade—The IL6 signaling cascade represents a promising therapeutic target to inhibit STAT3 activation [155,156,157,158,159,160,161,162]. However, while antibody-mediated approaches to neutralize IL6 (e.g., sirukumab and siltuximab) or block IL6R (e.g., tocilizumab) have demonstrated success in pre-clinical studies [158,168], phase I/II trials in patients with refractory/resistant NSCLC have yielded disappointing results (NCT00841191 [208], NCT00866970 [209,210]). Anti-IL6 treatment was also associated with numerous adverse side effects, including rectal hemorrhage, neutropenia, abnormal liver function, and increased risk of infections [208,209,210].

Inhibiting receptor tyrosine kinases (e.g., EGFR)—STAT3 is downstream of receptor tyrosine kinases such as EGFR, and persistent STAT3 activation due to aberrant EGFR signaling is well established in NSCLC [31,59]. Therapeutic strategies to block EGFR include monoclonal antibodies and receptor tyrosine kinase inhibitors. Monoclonal antibodies (e.g., necitumumab, matuzumab, panitumumab) target the extracellular domain of EGFR [224], while small molecules inhibitors (e.g., gefitinib, erlotinib) target the tyrosine kinase activity of EGFR [225,226]. However, despite the prevalence of EGFR mutations in NSCLC, many patients are refractory to EGFR-targeted therapies [227]. Several resistance mechanisms have been proposed and include secondary mutations in EGFR that prevent drug–receptor interactions and alternative mutations that result in compensatory increase in other oncogenic signaling pathways [209,228].

Inhibiting non-receptor tyrosine kinases (e.g., JAKs, SRC)—JAK inhibitors provide an effective means to decrease STAT3 activation and inhibit tumor growth. The JAK1/2-selective inhibitor ruxolitinib is approved for the treatment of myelofibrosis and polycythemia vera. In pre-clinical models of NSCLC, ruxolitinib decreases STAT3 activation, restores sensitivity to cisplatin chemotherapy, enhances apoptosis, and suppresses tumor growth [229,230,231]. However, ruxolitinib treatment has produced mixed results in NSCLC patients. A phase II trial of ruxolitinib (or placebo), pemetrexed, and cisplatin in patients with stage IIIb/IV or recurrent NSCLC showed good tolerability but was terminated without achieving an efficacy endpoint (NCT02119650 [212]). Likewise, ruxolitinib combined with the EGFR inhibitor erlotinib in a phase I/II study was well tolerated but did not achieve clinical benefit (NCT02155465). In contrast, a phase Ib study of ruxolitinib combined with afatinib (a second-generation EGFR targeting tyrosine kinase inhibitor) achieved partial response in 23% of patients and a disease control rate of 93% (NCT02145637 [211]). The combination was well tolerated, with no dose limiting toxicity observed. AZD1480 is another JAK1/2 tyrosine kinase inhibitor that has shown promising anti-tumor efficacy in an EGFR-driven model of lung adenocarcinoma by inhibiting STAT3 signaling [232,233] but lacked overall clinical activity in human NSCLC patients (NCT01219543 [214]). Of note, patients treated with JAK inhibitors also experienced severe adverse events, including thrombocytopenia, neutropenia, abnormal liver function, ataxia, and anemia [211,212,214].

Based on the importance of EGFR signaling in lung cancer, the known cooperation between EGFR and SRC proteins, and evidence of elevated SRC activity in human NSCLC, several studies have also evaluated the therapeutic effectiveness of SRC kinase inhibitors in lung cancer [57,234]. Treatment of A549 NSCLC cancer cells with small molecule SRC kinase inhibitors (e.g., PD180970 and SU6656) induced cell cycle arrest and tumor cell apoptosis by suppressing STAT3 activity [57]. Likewise, dasatinib selectively induced apoptosis in EGFR-mutant NSCLC cells, in part by blocking aberrant STAT3 activation [234]. However, treatment of NSCLC patients with dasatinib either alone [235], or in combination with EGFR inhibitors [219,220,221] has not resulted in clinical benefit.

### 6.2. Target SH2 Domain of STAT3

Two critical steps for STAT3 activation are the recognition of phosphotyrosine residues on cell surface receptors (which enable the binding of STAT3) and the recognition of the pY705 residue on another STAT3 molecule (which enables homodimerization). Both steps rely on the SH2 domain of STAT3, thereby making it an attractive therapeutic target to inhibit downstream STAT3 signaling.

Small molecule inhibitors that target the SH2 domain of STAT3 (e.g., C188-9, OPB-31121, OPB-51602, W2014-S, BBI-608) have demonstrated potent in vitro and in vivo anti-tumor activity in NSCLC models [70,236,237,238]. BBI-608 inhibits stemness gene expression, depletes CSCs, and overcomes cisplatin resistance in pre-clinical models of NSCLC [70], and showed encouraging signs of anti-cancer activity in a phase I/II study of NSCLC patients who received BBI-608 plus weekly paclitaxel (NCT01325441). A phase I study of OPB-51620 showed tumor regression in 2 (5%) patients with NSCLC (NCT01184807 [222]). The first responder exhibited complete regression of target lesions and remained progression-free for 6.9 months, while the other responder demonstrated a 41% reduction in tumor burden [222]. However, multiple treatment cycles with OPB-51620 was associated with systemic toxicities, including peripheral neuropathy, gastrointestinal distress, and lactic acidosis, which led to discontinuation of the study [222]. Severe side effects were also observed a phase I study of OPB-31121 in NSCLC patients and did not result in clinical benefit (NCT00955812 [223]). Thus, limitations of small molecule STAT3 inhibitors that target the SH2 domain include poor pharmacokinetic properties, insufficient potency, and non-specificity leading to severe adverse effects. Therapeutic targeting of the STAT3 SH2 domain may also be achieved via peptide inhibitors; however, limitations of this therapeutic strategy include poor cell permeability, selectivity, stability, and potential for adverse events due to improper stimulation of the immune system [239,240,241].

### 6.3. Promote Degradation of STAT mRNA

Anti-sense oligonucleotides bind to a complementary region of the target mRNA and inhibit gene expression by stimulating degradation of the mRNA via RNase [242]. However, as a class, antisense oligonucleotides have shown non-specific activation of the immune system due to the presence of unmethylated CpG motifs that are recognized as non-self [243]. Anti-sense oligonucleotides (e.g., AZD9150) against STAT3 are currently under clinical evaluation for the treatment of NSCLC (Table 1). Notably, initial studies using single-agent AZD9150 therapy demonstrated evidence of near complete resolution of highly treatment refractory NSCLC liver metastasis upon first re-staging, with additional stabilization of mediastinal lymph nodes in response to treatment [242].

### 6.4. Interfere with STAT3–DNA Binding

Once activated, STAT3 acts a transcription factor and binds to a response element in the promoter regions of target genes to induce gene expression. Thus, another approach to target the activity of STAT3 involves the use of double-stranded ‘decoy’ oligonucleotides (dsODN). These decoys closely mimic the STAT3 response element in the *c-fos* promoter and bind competitively to STAT3 to block the transcription of STAT3 target genes including BCL-xL and Cyclin D1 [244]. This approach has been used successfully to target STAT3 activation in NSCLC by enhancing tumor cell apoptosis [245,246]. A major limitation of early STAT3 decoys is the rapid degradation of the molecule by nucleases in the blood. Thus, a more stable circular oligonucleotide STAT3 decoy (CS3D) was developed with markedly enhanced thermal stability and a longer half-life in serum [247]. Notably, CS3D suppressed the growth of NSCLC xenografts [248] and prevented tobacco carcinogen-induced lung adenocarcinoma in mice [249]. These changes were accompanied by reduced tumor angiogenesis and fewer immunosuppressive immune cells [249].

G-quartet oligodeoxynucleotides (GQ-ODN) are another class of unique inhibitors that selectively target STAT3 dimers by inserting between their SH2 domains, resulting in STAT3 destabilization and reduced DNA binding ability [250,251]. Treatment of mice with GQ-ODN blocked the growth of NSCLC xenografts and significantly downregulated the expression of anti-apoptotic genes (e.g., BCL-2, BCL-xL, MCL-1), cell-cycle regulators (e.g., Cyclin D1 and c-MYC), and pro-angiogenic factors (e.g., VEGF) [250,251]. Despite these promising pre-clinical results, the selectivity of GQ-ODNs pose a potential concern since they exhibit a two- to four-fold greater IC_50_ for STAT1 over STAT3 [239]. Furthermore, due to their large size and charge, GQ-ODNs also exhibit poor membrane permeability. Thus, additional optimization is necessary for further development of clinical potential.

### 6.5. Challenges Associated with STAT3 Inhibition

Despite promising results from pre-clinical evaluation of anti-STAT3 inhibitors, the success of these drugs has not always successfully translated into a clinical setting. This is largely due to poor cell permeability, selectivity, and stability [239,240,241]. For example, STAT3 shares a significant level of homology with other family members such as STAT1, which plays a major role in defense against viral, mycobacterial, and fungal pathogens [252]. Thus, off-target STAT1 blockade by STAT3 inhibitors may increase vulnerability to infections by dampening anti-microbial immune responses and/or inhibition of IFN signaling. Similar systemic off-target effects have been reported following inhibition of the highly conserved SH2 domain of STAT3, which include neuropathy, gastrointestinal distress, and elevated blood pressure [222,223,239,240,241]. Thus, in addition to developing selective STAT3-targeting agents that do not affect the function of healthy cells, it will be important to recognize and adequately manage immune-related adverse effects to minimize treatment-related complications.

## 7. Therapeutic Opportunities

Although further research is required to maximize the translational success of STAT3-targeting therapies in the clinic, key opportunities include the use of STAT3 as a biomarker to predict drug response, as well as a therapeutic target to overcome drug resistance and enhance the efficacy of current NSCLC treatment modalities.

### 7.1. STAT3 as a Biomarker to Identify Patients with Aberrant EGFR Signaling

EGFR gene amplification and mutational status are important for predicting response to tyrosine kinase inhibitors [253]; however, identifying patients who are likely to derive clinical benefit remains a challenge. Detection of activated EGFR by immunohistochemistry in NSCLC patients is limited by the specificity of phospho-EGFR antibodies, since multiple tyrosine residues on EGFR can be phosphorylated [31]. Given that EGFR mutations correlate with an enriched STAT3 activation signature in human NSCLC and more than 50% of NSCLC tumors display activated EGFR-STAT3 signaling [31,59], phosphorylated STAT3 expression may be used as a biomarker to identify tumors with aberrant upstream EGFR signaling and stratify patients who are likely to benefit from EGFR inhibitors. Accordingly, high STAT3 expression is a predictor of clinical response to EGFR inhibition in EGFR-mutant patients [254]. This is further supported by studies demonstrating a key role of STAT3 in mediating oncogenic downstream effects of mutant EGFR, and conferring reduced sensitivity to EGFR inhibitors (e.g., gefitinib or erlotinib) [31,59,255]. For this reason, biomarker-driven clinical trials that co-target STAT3 and mutant EGFR warrant further investigation to improve the efficacy of EGFR inhibitors and prevent the emergence of drug resistance. To this end, synergistic anti-proliferative effects of EGFR and STAT3 inhibitors have been reported in pre-clinical models of NSCLC [256,257,258].

Most NSCLC patients that respond to EGFR inhibitors eventually develop progressive disease and become refractory to treatment [259]. While acquired resistance to EGFR inhibitors is typically associated with a second mutation in the EGFR kinase domain, the absence of a second mutation in some patients suggests that other drug resistance mechanisms may be involved [259,260]. Accordingly, phosphorylated STAT3 expression could also be used as a guide to measure EGFR activity and identify patients that may not necessarily have EGFR mutations, but still depend on EGFR signaling for tumor cell proliferation and survival.

### 7.2. Targeting STAT3 in Tyrosine Kinase Inhibitor Resistant NSCLC

EGFR tyrosine kinase inhibitors such as gefitinib and erlotinib are approved for first-line treatment of advanced NSCLC in patients with exon 19 deletions and an L858R point mutation in exon 21. While dramatic response rates are observed in some patients, most individuals eventually develop acquired drug resistance [227]. Approximately 50% of resistant cases exhibit a secondary mutation in the secondary T790M mutation in EGFR, which leads to the substitution of methionine for threonine in 790 [261,262]; however, the mechanism of resistance in the remaining population remains unclear.

Numerous studies have highlighted the therapeutic benefit of targeting STAT3 in NSCLC patients that are insensitive to current EGFR inhibitors [263]. A first-in-man phase I study of the oral small molecule STAT3 inhibitor OPB-51602 resulted in partial response in 5% of patients with EGFR-mutant NSCLC and resistance to EGFR tyrosine kinase inhibitors (NCT01184807) [222]. Consistent with previous findings demonstrating a link between STAT3 activation and treatment resistance to primary kinase inhibition in oncogene-addicted NSCLC [35], the therapeutic efficacy of OPB-51602 in NSCLC patients with tyrosine kinase drug resistance supports the potential for STAT3 as a drug target for patients with resistance to primary kinase inhibition.

It is also recognized that lung cancer cells can develop drug resistance through the compensatory increase in other oncogenic signaling pathways. This is observed in NSCLC, where long-term treatment with gefitinib suppresses EGFR/STAT3 signaling, but results in a compensatory increase in SRC/STAT3 and JAK2/STAT3 pathways that upregulate STAT3 activation [264]. Accordingly, increased STAT3 activity is observed in tumors of gefitinib-unresponsive NSCLC patients, while inhibition of STAT3 via siRNA-mediated interference in gefitinib-resistant NSCLC cells restores drug sensitivity and promotes apoptosis [264]. Meanwhile, treatment of tumor-bearing mice with the STAT3 inhibitor LL1 sensitizes resistant A549 NSCLC tumors to gefitinib and leads to a synergistic anti-tumor response [264,265]. These findings were corroborated in a separate study, where treatment of gefitinib- and erolotinib-resistant NSCLC with the STAT3 inhibitor W2014-S sensitized these cells to therapy in vitro and enhanced the anti-tumor efficacy of gefitinib in tyrosine kinase resistant lung cancer xenografts in vivo [238].

Constitutive activation of the phosphoinositide 3-kinase (PI3K)/AKT signaling pathway also drives tumor development and disease progression in NSCLC [266]. Several small molecule inhibitors targeting the PI3K/AKT signaling pathway are currently under pre-clinical investigation and in early phase clinical trials; however, many of these have yielded disappointing results [266]. Compensatory activation of MET/STAT3 signaling is observed following PI3K/AKT inhibition in NSCLC, and is proposed to be responsible for the reduced efficacy of PI3K/AKT inhibitors in clinical trials [267]. Accordingly, targeting the MET/STAT3 signaling pathway potentiates the anti-tumor efficacy of PI3K/AKT inhibitors in NSCLC tumor-bearing mice [266]. Meanwhile, inhibition of STAT3 with a selective inhibitor S3I-201 sensitizes refractory NSCLC cells to BEZ235 (a dual inhibitor of PI3K and mTOR) and enhances cell death [268]. These results collectively suggest that inhibition of STAT3 may represent an effective strategy to overcome resistance to PI3K/AKT/mTOR inhibition.

### 7.3. Targeting STAT3 to Overcome Chemo- and Radiotherapy Resistance

Aberrant activation of STAT3 plays a role in mediating chemoresistance in NSCLC, and over-expression of STAT3 is associated with cisplatin resistance in NSCLC cells [269,270]. Thus, STAT3 may represent a potential drug target to improve response to chemotherapy. The anthracycline antibiotic doxorubicin is widely used for the treatment of lung cancer [271]; however, in contrast to its efficacy in SCLC, less than 50% of NSCLC patients respond to therapy [272]. Strikingly, treatment of mice harboring H1650 NSCLC xenografts with the STAT3 inhibitor RITA in combination with doxorubicin significantly reduces tumor growth compared to monotherapy-treated groups [263]. In other studies, siRNA-mediated STAT3 inhibition enhanced the sensitivity of A549 and SPC-A1 NSCLC cells to cisplatin by enhancing apoptosis in a caspase-3-dependent manner [33], while the small molecule STAT3 inhibitor BBI-608 re-sensitized chemo-resistant NSCLC cells to cisplatin and inhibited cell proliferation [70]. Pharmacologic inhibition of STAT3 via administration of C188-9 also improved the response of NSCLC xenografts to cisplatin and vinblastine, and significantly reduced tumor growth in the combination-treated group compared to mice treated with single agent chemotherapies [273]. Meanwhile, treatment of EGFR-mutant NSCLC cells with the STAT3 inhibitor TPCA-1 enhanced the anti-proliferative effects of afatinib chemotherapy [274]. Together, these findings support the use of STAT3 inhibitors as a complementary strategy to improve response of NSCLC to chemotherapy.

Radiotherapy is commonly used to treat early stage inoperable NSCLC [275] and locally advanced disease [276,277]; however, the emergence of radioresistant cells remains a significant therapeutic obstacle [278]. Exposure of human NSCLC cells to ionizing radiation results in the activation of JAK2/STAT3 and the upregulation of BCL-2/BCL-xL cell survival signaling pathways, which are also persistently activated in human NSCLC cells with acquired radio-resistance [279]. Accordingly, inhibition of STAT3 by niclosamide reverses acquired radio-resistance of NSCLC cancer xenografts and improves tumor shrinkage, while shRNA-mediated knock-down of STAT3 restores sensitivity of lung cancer cells to ionizing radiation [34,279]. Collectively, these results support STAT3 as a molecular target for improving radiotherapy-sensitization of NSCLC.

### 7.4. Combining STAT3 Inhibition with Immune Checkpoint Blockade

The PD1/PDL1 pathway is an important immune checkpoint that regulates self-tolerance and restrains excessive immune responses in normal tissues [280]. PD1 is expressed on immune cells, including activated T-cells, while PDL1 and PDL2 are expressed on antigen-presenting cells and non-immune cells in peripheral tissues [281]. Engagement of PD1 with PDL1 suppresses cytotoxic T-cell responses by inducing apoptosis, immune cell anergy, and the production of immunosuppressive cytokines [281]. However, this interaction is reversible and blockade of PD1/PDL1 interactions restores T-cell function. Accordingly, the PD1/PDL1 signaling axis is often hijacked by cancer cells to evade immune-mediated tumor surveillance, while inhibition of this pathway enhances tumor cell death [282]. Of note, elevated levels of PDL1 are observed in NSCLC patients [283,284,285,286], and PD1/PDL1 checkpoint inhibitors have become routinely part of the clinical approach for management of NSCLC [287,288,289,290].

Although PDL1 expression is increased in oncogene-addicted NSCLC, monotherapy with immune checkpoint inhibitors have not produced encouraging results [291,292]. These findings highlight the need for further investigation into the combined use of STAT3 inhibitors with immune checkpoint blockade, since STAT3 is directly related to the expression of PDL1 in EGFR-mutant NSCLC [80], as well as in tumors that display chromosomal arrangements of ALK [293,294,295,296]. STAT3 is also partially involved in the control of ectopic PDL1 expression in KRAS-mutant NSCLC, and can be targeted to reduce PDL1 expression [297]. Meanwhile, the microRNA-3127-5p promotes STAT3 activation and induces the expression of PDL1 to facilitate immune escape and chemoresistance [298]. In gefitinib-resistant NSCLC cells, inhibition of STAT3 reduces PDL1 expression [299], and enhances the efficacy of anti-PD1/PDL1 immunotherapies in NSCLC tumor-bearing mice [81].

Patients with non-squamous STK11-mutant NSCLC are also less likely to respond to combined anti-PD1 anti-CTLA4 immune checkpoint blockade than STK11 wild-type patients [300]. This is attributed to an increased expression of genes and cytokines that activate STAT3 signaling, since inhibition of STAT3 signaling via an anti-sense oligonucleotide reversed immunotherapy resistance in pre-clinical STK11 knock-out models [300]. In line with these findings, treatment of non-squamous NSCLC cell lines with pemetrexed chemotherapy leads to enhanced PDL1 expression through activation of mTOR and STAT3 signaling pathways [301].

Furthermore, tumor cell-extrinsic STAT3 activation also regulates the immune tolerance and suppression of anti-tumor immunity by upregulating the expression of immune checkpoint proteins (e.g., PD1, CTLA4) on the surface of tumor-associated myeloid cells [92], B-cells [123], Tregs [134], and CD4 and CD8 T-cells [144,145]. Collectively, these findings suggest a role for tumor cell-intrinsic and -extrinsic STAT3 signaling in regulating anti-tumor immunity via expression of checkpoint molecules and represent a promising strategy to improve the efficacy of immune checkpoint inhibitors. To this end, combined STAT3 inhibitors such as anti-sense oligonucleotides (AZD9150) [242] are currently undergoing clinical evaluation in combination with anti-PD1 immunotherapies for the treatment of NSCLC (NCT03334617, NCT02983578).

## 8. Concluding Remarks

Given the multifaceted role of STAT3 in promoting NSCLC tumorigenesis and its association with a poor prognosis in human patients, therapeutic modalities based on STAT3 inhibition will have widespread therapeutic applications. To date, strategies aimed at targeting STAT3 signaling include blocking upstream signaling interactions, inhibiting the SH2 domain of STAT3, promoting the degradation of STAT3 mRNA, and interfering with STAT3 DNA binding. Some of these drugs also demonstrate complementary anti-tumor effects when combined with chemotherapy, tyrosine kinase inhibitors, and immunotherapy, suggesting the additive benefit of co-targeting STAT3 in these settings. Thus, further research is required to improve the pharmacokinetics, potency, and selectivity of STAT3-targeting drugs in order to realize their ultimate potential in the clinic.

## Figures and Tables

**Table 1:** Clinical trials assessing STAT3 inhibition in NSCLC.

Mechanism	Drug	Phase	Toxicities	Responses	Outcome	References
Inhibit IL6/JAK/STAT3 signaling	Siltuximab ^a^	I/II	Hepatic function abnormalities, neutropenia	No objective responses	Completed	NCT00841191 [208]
	ALD518 ^a^	II	No dose limiting toxicity	Reduction of anemia and cachexia	Completed	NCT00866970 [209,210]
Inhibit receptor/non-receptor tyrosine kinases	Ruxolitinib ^b^ + Afatinib ^c^	I	Diarrhea, anemia, paronychia, acneiform rash, oral mucositis	ORR 23.3% Median PFS 4.9 months	Completed	NCT02145637 [211]
	Ruxolitinib ^b^ + Pemetrexed/Cisplatin ^d^	II	No dose limiting toxicity with the combination	Response rate: 31% (ruxolitinib) vs. 35% (placebo)	Terminated (no clinical benefit)	NCT02119650 [212]
	Ruxolitinib ^b^ + Erlotinib ^c^	I/II	Anemia, diarrhea, liver function derangement	ORR 5% Median PFS 2.2 months	Completed	NCT02155465 [213]
	AZD1480 ^b^	I	Pleiotropic neurologic toxicity	No responses seen	Completed	NCT01219543 [214]
	AZD4205 ^b^ + Osimertinib ^c^	I/II	Not reported		Completed	NCT03450330
	Itacitinib ^b^ + Pembrolizumab ^e^	II			Ongoing, N/A	NCT03425006
	Itacitinib^2^ + Osimertinib ^c^	I/II			Ongoing, N/A	NCT02917993
	Momelotinib ^b^ + Trametinib ^c^	I	GI toxicity, fatigue, liver function derangement	No objective responses Median PFS 3.6 months	Terminated (no clinical benefit)	NCT02258607 [215]
	Momelotinib ^b^ + Erlotinib ^c^	I	Neutropenia, diarrhea, skin toxicity, fatigue	ORR 54.5% Median PFS 9.2 months	Terminated (no benefit over erlotinib monotherapy)	NCT02206763 [216]
	Pacritinib ^b^ + Erlotinib ^c^	I/II	Not reported		Terminated (due to drug shortage)	NCT02342353
	Dasatinib ^f^	II	Fatigue, dyspnea	ORR 3%	Completed	NCT00459342 [217]
	Dasatinib ^f^	II	≥Grade 3 toxicity: dyspnea, fatigue, AST elevation, anorexia, nausea		Terminated (safety concerns)	NCT01491633 [218]
	Dasatinib ^f^ + Afatinib ^c^	I	New or increased pleural effusions	No objective responses	Completed	NCT01999985 [219]
	Dasatinib ^f^ + Osimertinib ^c^	I	Pleural effusions, myelosuppression, rash, transaminitis	ORR: 90% Median PFS 19.4 months	Completed (prematurely closed due to slow accrual)	NCT02954523 [220]
	Dasatinib ^f^ + Erlotinib ^c^	I/II	Not reported	ORR: 15% Median PFS 3.3 months	Completed	NCT00826449 [221]
Block STAT3 dimerization	OPB-51620 ^g^	I	Fatigue, GI toxicity, early-onset peripheral neuropathy		Completed	NCT01184807 [222]
	OPB-31121 ^g^	I	GI toxicity	No objective responses	Completed	NCT00955812 [223]
	C188-9 (TTI-101) ^g^	I			Recruiting	NCT03195699
Promote degradation of STAT3 mRNA	AZD9150 ^h^ + anti-PDL1 ^e^	II			Ongoing, N/A	NCT02983578
	AZD9150 ^h^ + anti-PDL1 ^e^	II			Ongoing, N/A	NCT03334617
	AZD9150 ^h^ + anti-PDL1 ^e^	I/II			Ongoing, N/A	NCT03421353

^a^ Anti-IL6 antibody; AST: aspartate transaminases; GI: gastrointestinal; ^b^ JAK inhibitor; ^c^ EGFR inhibitor; ^d^ chemotherapy; ^e^ immunotherapy; ORR: objective response rate; PFS: progression free survival; ^f^ SRC kinase inhibitor; ^g^ STAT3 small molecule inhibitor; ^h^ STAT3 anti-sense oligonucleotide.
